# Supplementary figures and images for: Transcriptional Inflammatory Signature in Healthy Donors and Different Radiotherapy Cancer Patients
Source: Int J Mol Sci. 2024 Jan 16;25(2):1080. doi: 10.3390/ijms25021080 (PMC10816540; doi:10.3390/ijms25021080)

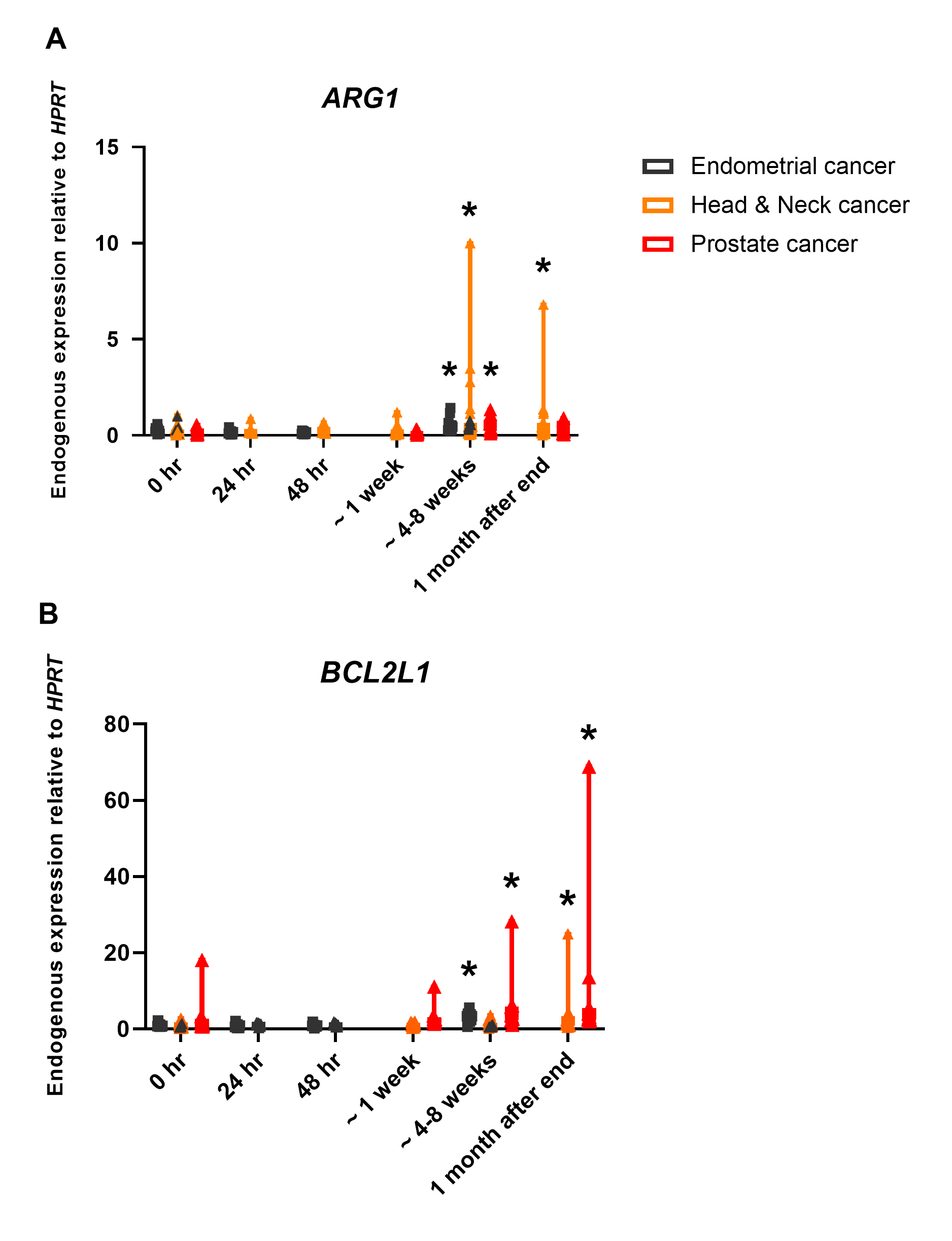

Supplement: Supplementary file 1 [file ijms-25-01080-s001.zip › Supplementary Figure S1.tif]

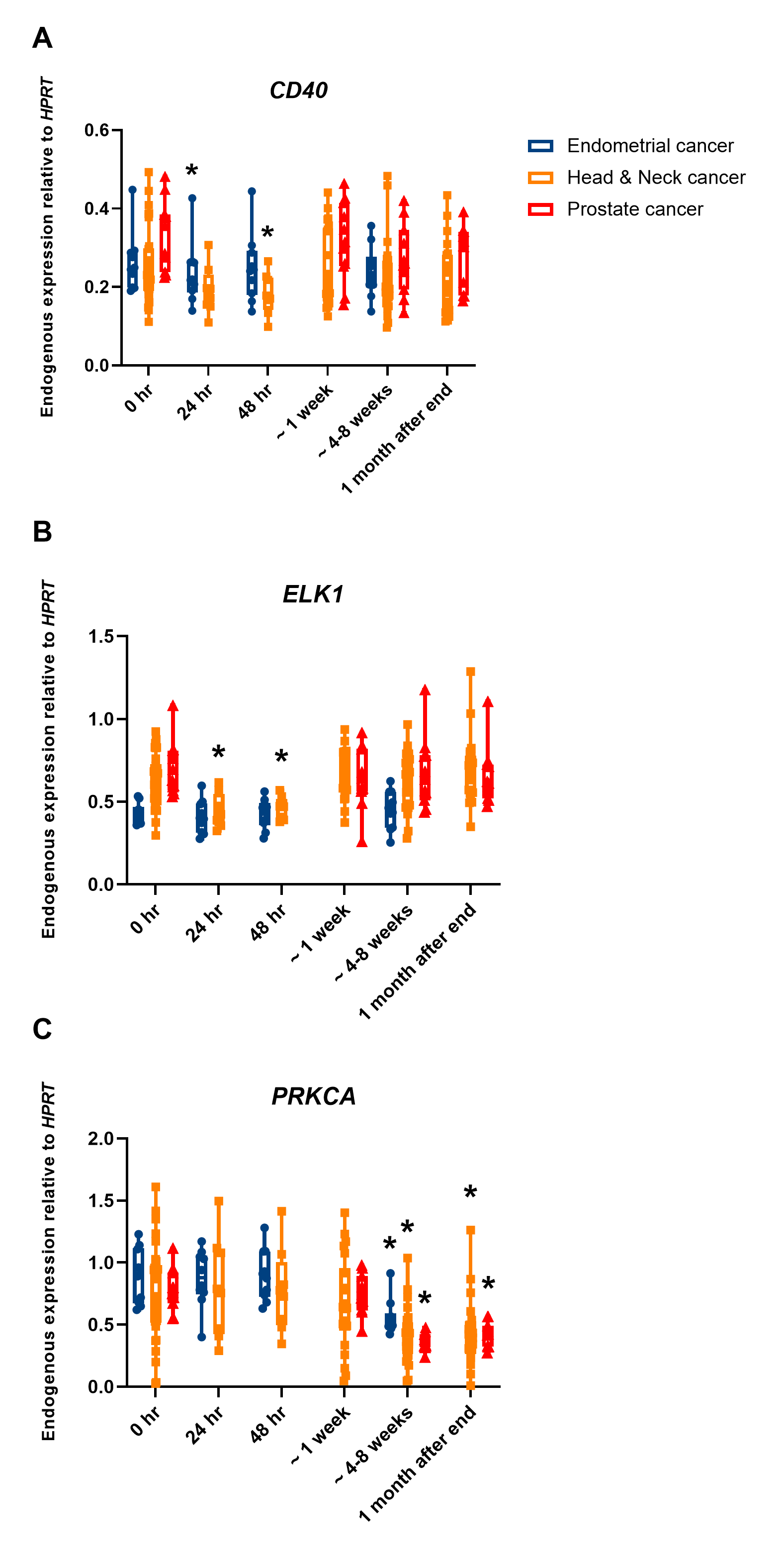

Supplement: Supplementary file 1 [file ijms-25-01080-s001.zip › Supplementary Figure S2.tif]

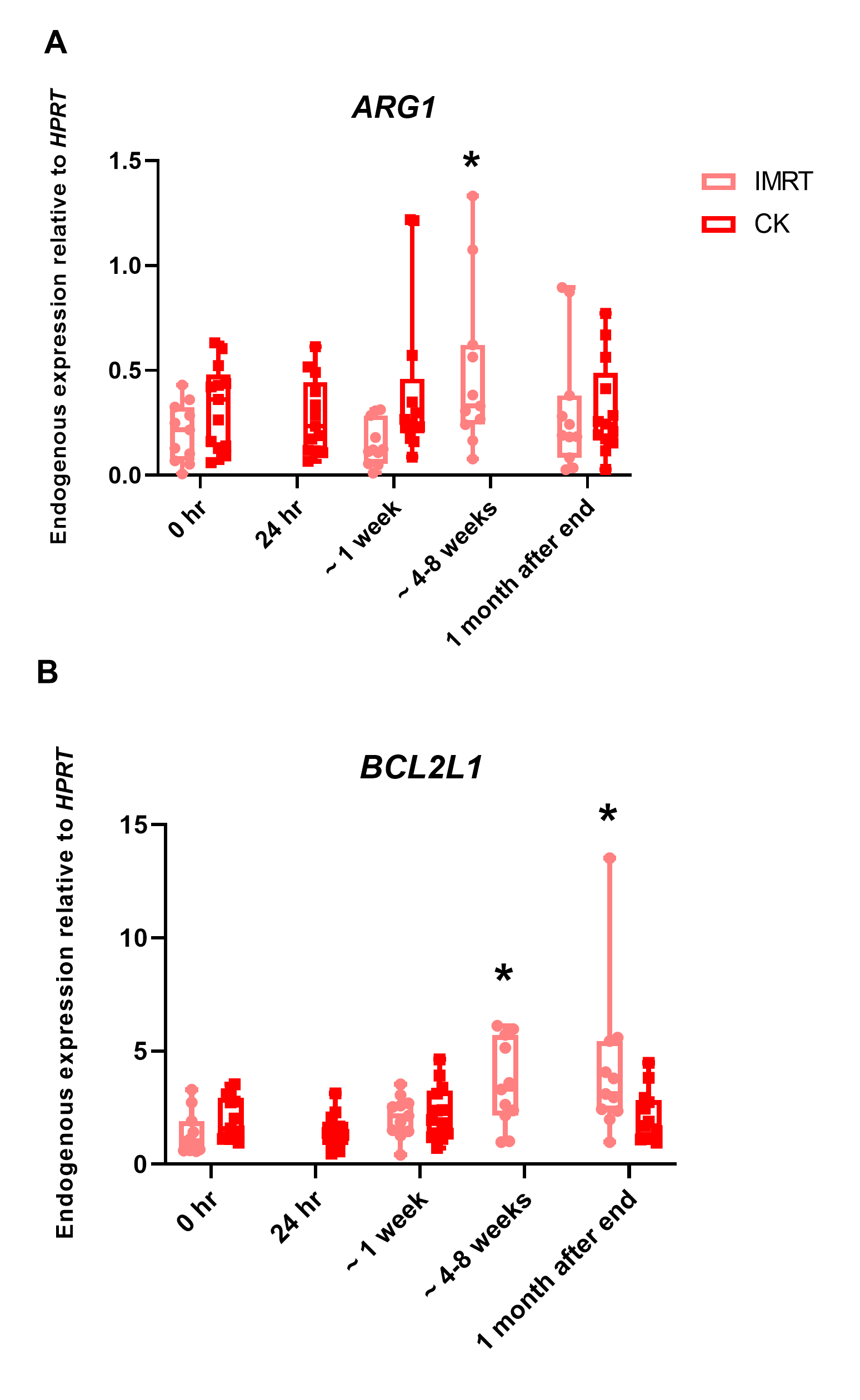

Supplement: Supplementary file 1 [file ijms-25-01080-s001.zip › Supplementary Figure S3.tif]

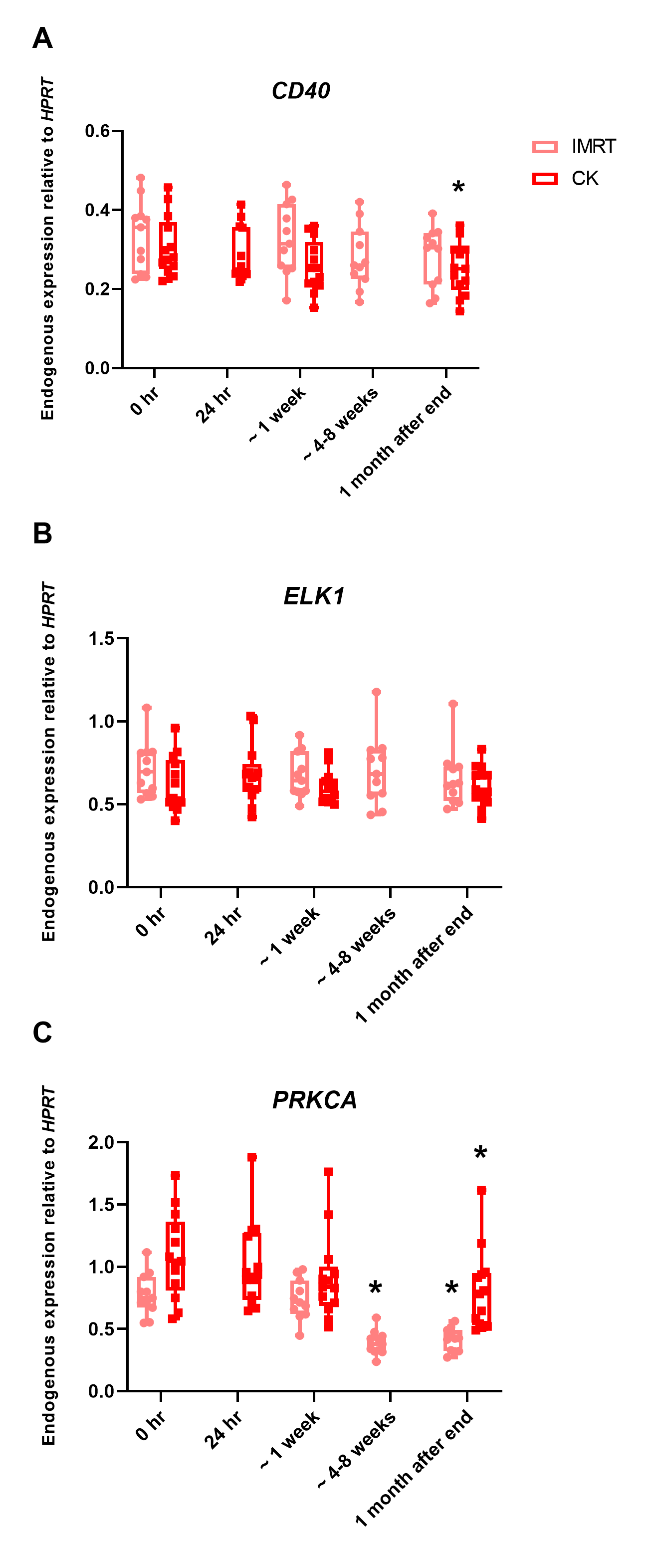

Supplement: Supplementary file 1 [file ijms-25-01080-s001.zip › Supplementary Figure S4.tif]
